# The genome sequence of the garden bumblebee, *Bombus hortorum *(Linnaeus, 1761)

**DOI:** 10.12688/wellcomeopenres.17187.1

**Published:** 2021-10-14

**Authors:** Liam Crowley, University of Oxford and Wytham Woods Genome Acquisition Lab, Darwin Tree of Life Barcoding collective, Wellcome Sanger Institute Tree of Life programme, Wellcome Sanger Institute Scientific Operations: DNA Pipelines collective, Tree of Life Core Informatics collective, Darwin Tree of Life Consortium

**Affiliations:** 1 Department of Zoology, University of Oxford, Oxford, OX1 3SZ, UK

**Keywords:** Bombus hortorum, garden bumblebee, genome sequence, chromosomal

## Abstract

We present a genome assembly from an individual female *Bombus hortorum *(the garden bumblebee; Arthropoda; Insecta; Hymenoptera; Apidae). The genome sequence is 296 megabases in span. The majority of the assembly is scaffolded into 18 chromosomal pseudomolecules.

## Species taxonomy

Metazoa; Arthropoda; Insecta; Endopterygota; Hymenoptera; Apocrita; Aculeata; Apoidea; Apidae; Apinae; Bombini; Bombus; Megabombus; *Bombus hortorum* Linnaeus 1761 (NCBI:txid85660).

## Introduction

The garden bumblebee, *Bombus hortorum*, is one of the seven most common species of bumblebee in the UK. It is widespread, being found in most habitats apart from upland areas. It is a large bumblebee species with a long face and a very long proboscis, meaning it favours deep flowers with a relatively long corolla such as foxglove ( *Digitalis purpurea*) and honeysuckle *(Lonicera periclymenum*). It visits a wide range of flowers, particularly those with deep or complex blooms, meaning it is frequently found in gardens. This species expresses a preference for red clover ( *Trifolium pratense*) when available ( Brown *et al*., 1992). In common with other species in the Genus, the high degree of floral visitation undertaken by this species indicates its important role as a pollinator.

*Bombus hortorum* is a eusocial, annual species, with queens emerging from overwinter diapause from around March onwards. Workers can be seen from late April, with the number of workers increasing throughout the spring to reach around 100 workers in a mature nest ( Edwards & Jenner, 2005). The first males can be produced from June and new queens from July. Nests are always constructed under cover, but if underground they are often only shallowly so ( Kells & Goulson, 2003).

## Genome sequence report

**Figure 1.**
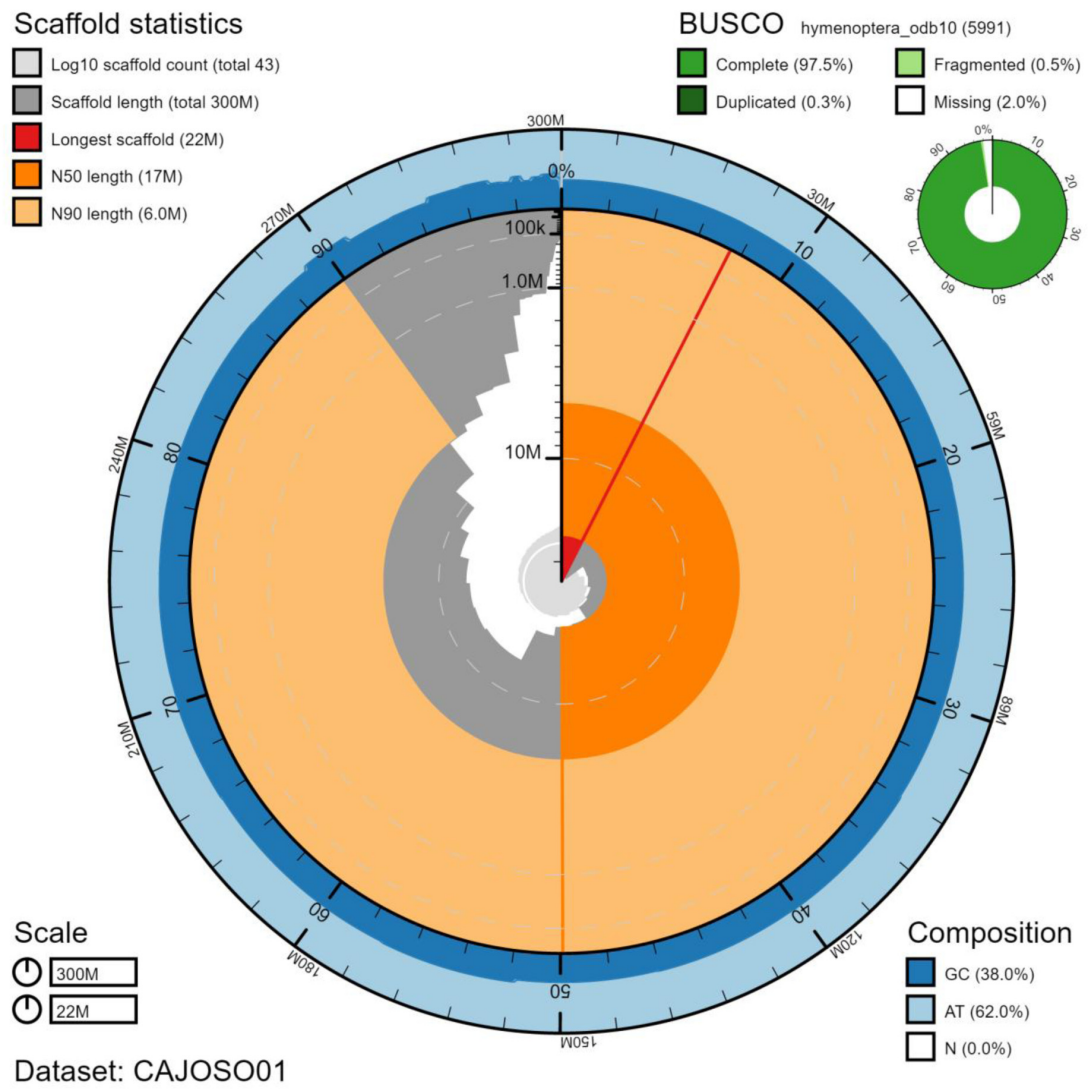
Genome assembly of *Bombus hortorum*, iyBomHort1.1: metrics. The BlobToolKit Snailplot shows N50 metrics and BUSCO gene completeness. An interactive version of this figure is available at https://blobtoolkit.genomehubs.org/view/iyBomHort1.1/dataset/CAJOSO01/snail.

The genome was sequenced from a single female *B. hortorum* collected from Wytham Woods, Oxfordshire, UK (latitude 51.77, longitude -1.339). A total of 82-fold coverage in Pacific Biosciences single-molecule long reads and 113-fold coverage in 10X Genomics read clouds were generated. Primary assembly contigs were scaffolded with chromosome conformation Hi-C data. Manual assembly curation corrected 23 missing/misjoins, reducing the assembly length by 0.001% and the scaffold number by 31.1%, and increasing the scaffold N50 by 38.9%. The final assembly has a total length of 296 Mb in 43 sequence scaffolds with a scaffold N50 of 17 Mb ( Table 1). Of the assembly sequence, 88.9% was assigned to 18 chromosomal-level scaffolds (numbered by sequence length) ( Figure 1– Figure 4; Table 2). The assembly has a BUSCO ( Simão *et al.*, 2015) v5.1.2 completeness of 97.5% using the hymenoptera_odb10 reference set. While not fully phased, the assembly deposited is of one haplotype. Contigs corresponding to the second haplotype have also been deposited.

**Table 1.** Genome data for *Bombus hortorum*, iyBomHort1.1.

*Project accession data*	
Assembly identifier	iyBomHort1
Species	*Bombus hortorum*
Specimen	iyBomHort1
NCBI taxonomy ID	NCBI:txid85660
BioProject	PRJEB43539
BioSample ID	SAMEA7520483
Isolate information	Female, head/thorax/abdomen
*Raw data accessions*	
PacificBiosciences SEQUEL II	ERR6054540-ERR6054544, ERR6548407
10X Genomics Illumina	ERR6054540-ERR6054543
Hi-C Illumina	ERR6054544
RNAseq PolyA Illumina	ERR6001535
*Genome assembly*	
Assembly accession	GCA_905332935.1
*Accession of alternate haplotype*	GCA_905333095.1
Span (Mb)	296
Number of contigs	73
Contig N50 length (Mb)	11
Number of scaffolds	43
Scaffold N50 length (Mb)	17
Longest scaffold (Mb)	22
BUSCO * genome score	C:97.5%[S:97.3%,D:0.3%],F:0.5 %,M:2.0%,n:5991

*BUSCO scores based on the hymenoptera_odb10 BUSCO set using v5.1.2. C= complete [S= single copy, D=duplicated], F=fragmented, M=missing, n=number of orthologues in comparison. A full set of BUSCO scores is available at https://blobtoolkit.genomehubs.org/view/iyBomHort1.1/dataset/CAJOSO01/busco.

**Figure 2.**
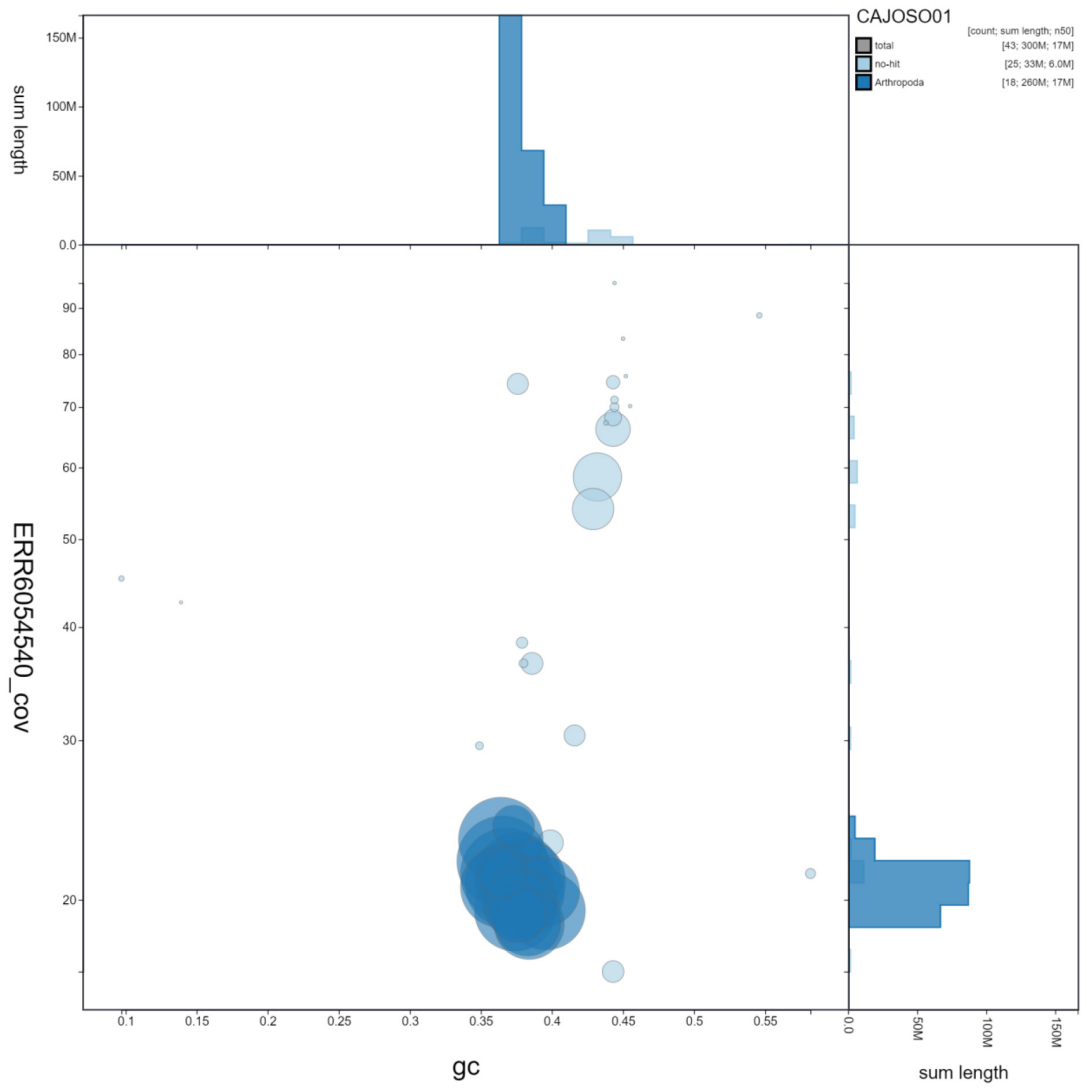
Genome assembly of *Bombus hortorum*, iyBomHort1.1: GC coverage. BlobToolKit GC-coverage plot. Scaffolds are coloured by phylum. Circles are sized in proportion to scaffold length. Histograms show the distribution of scaffold length sum along each axis. An interactive version of this figure is available at https://blobtoolkit.genomehubs.org/view/iyBomHort1.1/dataset/CAJOSO01/blob.

**Figure 3.**
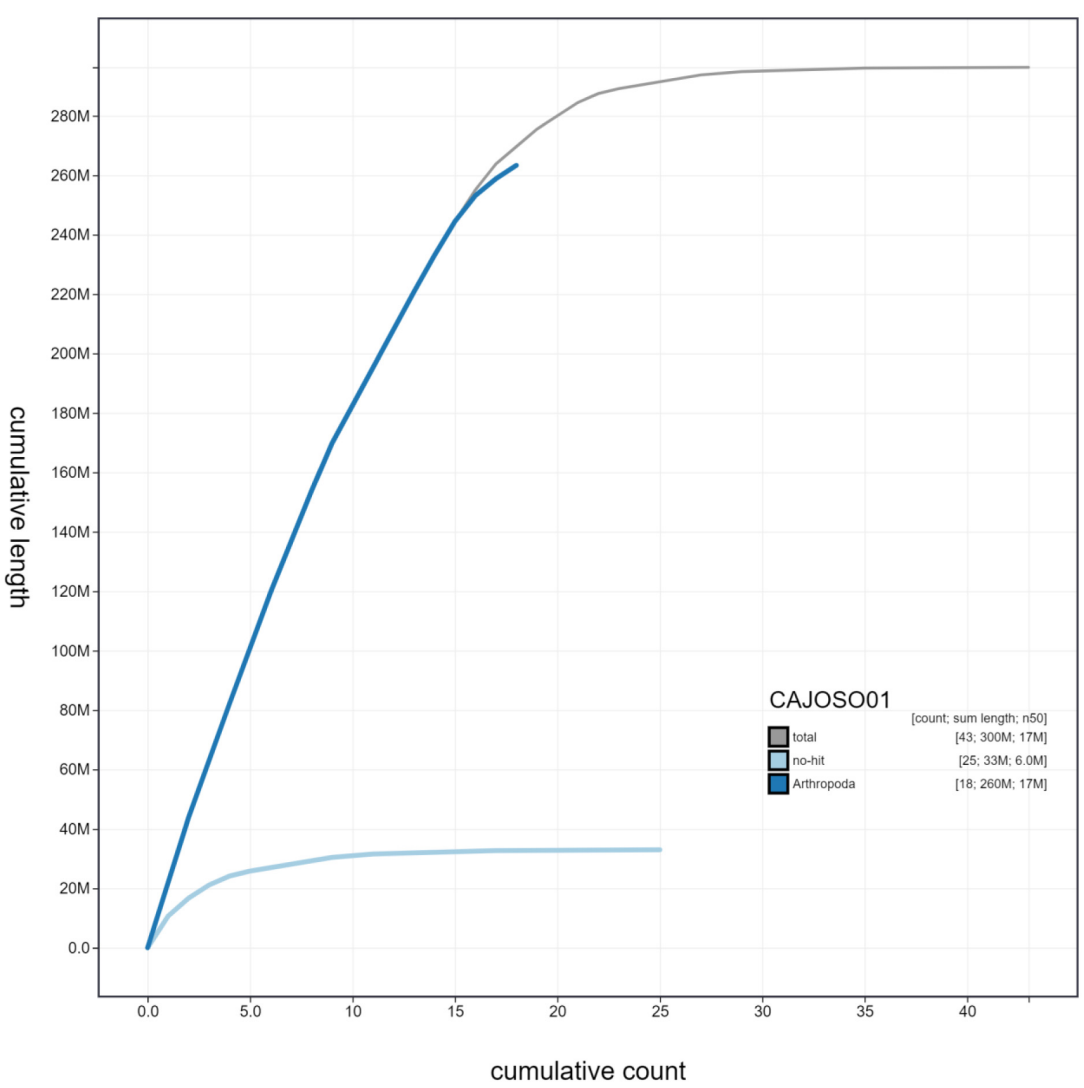
Genome assembly of *Bombus hortorum*, iyBomHort1.1: cumulative sequence. BlobToolKit cumulative sequence plot. The grey line shows cumulative length for all chromosomes. Coloured lines show cumulative lengths of chromosomes assigned to each phylum using the buscogenes taxrule. An interactive version of this figure is available at https://blobtoolkit.genomehubs.org/view/iyBomHort1.1/dataset/CAJOSO01/cumulative.

**Figure 4.**
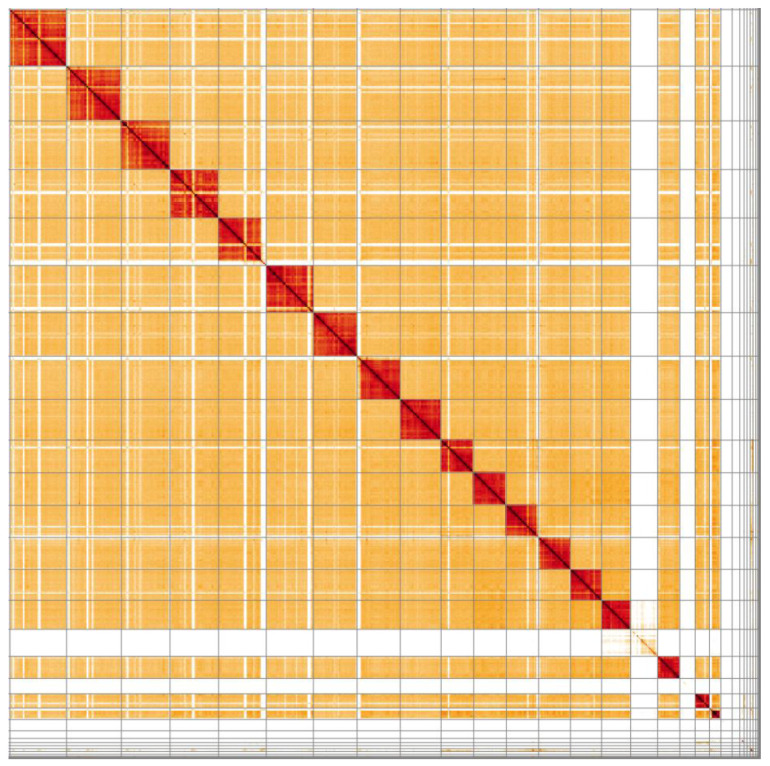
Genome assembly of *Bombus hortorum*, iyBomHort1.1: Hi-C contact map. Hi-C contact map of the iyBomHort1.1 assembly, visualised in HiGlass.

**Table 2.** Chromosomal pseudomolecules in the genome assembly of *Bombus hortorum*, iyBomHort1.1.

INSDC accession	Chromosome	Size (Mb)	GC%
HG995188.1	1	22.27	36.8
HG995189.1	2	21.59	36.5
HG995190.1	3	19.19	37.6
HG995191.1	4	19.11	36.9
HG995192.1	5	18.77	36.4
HG995193.1	6	18.59	38
HG995194.1	7	17.19	37.4
HG995195.1	8	17.02	36.4
HG995196.1	9	16.08	39.6
HG995197.1	10	12.86	38.4
HG995198.1	11	12.85	38.4
HG995199.1	12	12.68	39.5
HG995200.1	13	12.56	37.9
HG995201.1	14	12.25	37.6
HG995202.1	15	11.45	38.3
HG995203.1	16	8.71	37.6
HG995204.1	17	5.65	36.5
HG995205.1	18	4.51	37.3
HG995206.1	MT	0.02	14.2
-	Unplaced	32.96	41.3

## Methods

A single female *B. hortorum* was collected using a net from Wytham Woods, Oxfordshire, UK (latitude 51.77, longitude -1.339) by Liam Crowley, University of Oxford. The specimen was snap-frozen in dry ice using a CoolRack before transferring to the Wellcome Sanger Institute (WSI) for genome sequencing and assembly.

DNA was extracted at the WSI Scientific Operations core from the head and thorax using the Qiagen MagAttract HMW DNA kit, according to the manufacturer’s instructions. RNA was extracted from abdomen tissue in the Tree of Life Laboratory at the WSI using TRIzol (Invitrogen), according to the manufacturer’s instructions. RNA was then eluted in 50 μl RNAse-free water and its concentration RNA assessed using a Nanodrop spectrophotometer and Qubit Fluorometer using the Qubit RNA Broad-Range (BR) Assay kit. Analysis of the integrity of the RNA was done using Agilent RNA 6000 Pico Kit and Eukaryotic Total RNA assay.

Pacific Biosciences HiFi circular consensus and 10X Genomics read cloud sequencing libraries, in addition to PolyA RNA-Seq libraries, were constructed according to the manufacturers’ instructions. Sequencing was performed by the Scientific Operations core at the Wellcome Sanger Institute on Pacific Biosciences SEQUEL II (HiFi), Illumina HiSeq X (10X) and Illumina HiSeq 4000 (RNA-Seq) instruments. Hi-C data were generated from head and thorax tissue using the Arima v2.0 kit and sequenced on HiSeq X.

Assembly was carried out with Hifiasm ( Cheng *et al.*, 2021). Haplotypic duplication was identified and removed with purge_dups ( Guan *et al.*, 2020). The assembly was polished with the 10X Genomics Illumina data by aligning to the assembly with longranger align, calling variants with freebayes ( Garrison & Marth, 2012). One round of the Illumina polishing was applied. Scaffolding with Hi-C data ( Rao *et al.*, 2014) was carried out with SALSA2 ( Ghurye *et al.*, 2019). The assembly was checked for contamination and corrected using the gEVAL system ( Chow *et al.*, 2016) as described previously ( Howe *et al.*, 2021). Manual curation was performed using gEVAL, HiGlass ( Kerpedjiev *et al.*, 2018) and Pretext. The mitochondrial genome was assembled using MitoHiFi ( Uliano-Silva *et al.*, 2021). The genome was analysed and BUSCO scores generated within the BlobToolKit environment ( Challis *et al.*, 2020). Table 3 contains a list of all software tool versions used, where appropriate.

**Table 3.** Software tools used.

Software tool	Version	Source
Hifiasm	0.12	Cheng *et al.*, 2021
purge_dups	1.2.3	Guan *et al.*, 2020
SALSA2	2.2	Ghurye *et al.*, 2019
longranger align	2.2.2	https://support.10xgenomics.com/genome-exome/ software/pipelines/latest/advanced/other-pipelines
freebayes	1.3.1-17-gaa2ace8	Garrison & Marth, 2012
MitoHiFi	1.0	Uliano-Silva *et al.*, 2021
gEVAL	N/A	Chow *et al.*, 2016
HiGlass	1.11.6	Kerpedjiev *et al.*, 2018
PretextView	0.1.x	https://github.com/wtsi-hpag/PretextView
BlobToolKit	2.6	Challis *et al.*, 2020

The materials that have contributed to this genome note have been supplied by a Darwin Tree of Life Partner. The submission of materials by a Darwin Tree of Life Partner is subject to the Darwin Tree of Life Project Sampling Code of Practice. By agreeing with and signing up to the Sampling Code of Practice, the Darwin Tree of Life Partner agrees they will meet the legal and ethical requirements and standards set out within this document in respect of all samples acquired for, and supplied to, the Darwin Tree of Life Project. Each transfer of samples is further undertaken according to a Research Collaboration Agreement or Material Transfer Agreement entered into by the Darwin Tree of Life Partner, Genome Research Limited (operating as the Wellcome Sanger Institute), and in some circumstances other Darwin Tree of Life collaborators.

## Data availability

European Nucleotide Archive: Bombus hortorum (garden bumblebee). Accession number PRJEB43539: https://identifiers.org/ena.embl:PRJEB43539

The genome sequence is released openly for reuse. The *B. hortorum* genome sequencing initiative is part of the Darwin Tree of Life (DToL) project. All raw sequence data and the assembly have been deposited in INSDC databases. The genome will be annotated using the RNA-Seq data and presented through the Ensembl pipeline at the European Bioinformatics Institute. Raw data and assembly accession identifiers are reported in Table 1.
