# Supplementary material for: The genome sequence of the garden bumblebee, Bombus hortorum (Linnaeus, 1761)
Source: Wellcome Open Res. 2021 Oct 14;6:270. [Version 1] doi: 10.12688/wellcomeopenres.17187.1 (PMC8543170; doi:10.12688/wellcomeopenres.17187.1)
Supplement: Peer review history

# Peer review: Reviewer response for version 1

**DOI:** 10.21956/wellcomeopenres.18990.r46472

**Published:** 2021-10-29

**Authors:** Susan Brown

**Affiliations:** 1 KSU Bioinformatics Center, Division of Biology, Kansas State University, Manhattan, KS, USA

This report on the genome assembly for the garden bumblebee is concise, complete and clearly written. As with all Tree of LIfe projects, DNA from a single female individual was sequenced using PacBio long read and scaffolding with Hi-C reads. The Blobtools kit provides an easily understandable graphic summary of the assembly. The BUSCO score of 97.5% indicates a predominantly complete genome representation. This assembly is in excellent shape for annotation and downstream analysis.

Are sufficient details of methods and materials provided to allow replication by others?

Yes

Is the rationale for creating the dataset(s) clearly described?

Yes

Are the datasets clearly presented in a useable and accessible format?

Yes

Are the protocols appropriate and is the work technically sound?

Yes

Reviewer Expertise:

Genomics, bioinformatics and Deelopmental Genetics of the red flour beetle.

I confirm that I have read this submission and believe that I have an appropriate level of expertise to confirm that it is of an acceptable scientific standard.

# Peer review: Reviewer response for version 1

**DOI:** 10.21956/wellcomeopenres.18990.r46468

**Published:** 2021-10-22

**Authors:** Robert Waterhouse

**Affiliations:** 1 Department of Ecology and Evolution, University of Lausanne, Lausanne, Switzerland

This Data Note presents a clear and comprehensive description of all the steps taken to generate the *Bombus hortorum* genome assembly spanning 296 Mb with 90% assigned to 18 chromosomal-level scaffolds. As a member of an important group of pollinators, the rationale for building these resources is clear. The described data collection and analysis methods follow the best practices in the field and have delivered a high-quality complete and accurate chromosome-level reference genome.

Are sufficient details of methods and materials provided to allow replication by others?

Yes

Is the rationale for creating the dataset(s) clearly described?

Yes

Are the datasets clearly presented in a useable and accessible format?

Yes

Are the protocols appropriate and is the work technically sound?

Yes

Reviewer Expertise:

Arthropod genomics

I confirm that I have read this submission and believe that I have an appropriate level of expertise to confirm that it is of an acceptable scientific standard.

# Peer review: Reviewer response for version 1

**DOI:** 10.21956/wellcomeopenres.18990.r46470

**Published:** 2021-10-19

**Authors:** Stephen Richards

**Affiliations:** 1 Human Genome Sequencing Center, Department of Human and Molecular Genetics, Baylor College of Medicine, Houston, TX, USA

The authors present a truly excellent extremely high-quality genome reference of *Bombus hortorum*. The reference genome is high quality enough that it will stand the test of time, and likely be the go-to reference for at least the next 100 years, and possibly much longer.

 In terms of data access, I was able to easily find and download the sequence from my INSDC database (NCBI) and had no trouble blasting my favorite gene against it (something that not all genome data providers always do, and so to be congratulated here.). Not really sure there is anything else I can add for a review of a data note, the data has been very well generated in a transparent manner and very well made globally available.

Are sufficient details of methods and materials provided to allow replication by others?

Yes

Is the rationale for creating the dataset(s) clearly described?

Yes

Are the datasets clearly presented in a useable and accessible format?

Yes

Are the protocols appropriate and is the work technically sound?

Yes

Reviewer Expertise:

Arthropod genomics and genomics in general.

I confirm that I have read this submission and believe that I have an appropriate level of expertise to confirm that it is of an acceptable scientific standard.
